# Immunotherapy as a Neoadjuvant Therapy for a Patient with Hepatocellular Carcinoma in the Pretransplant Setting: A Case Report

**DOI:** 10.3390/curroncol29060341

**Published:** 2022-06-15

**Authors:** Maen Abdelrahim, Abdullah Esmail, Godsfavour Umoru, Kiersten Westhart, Ala Abudayyeh, Ashish Saharia, Rafik M. Ghobrial

**Affiliations:** 1 Section of GI Oncology, Department of Medical Oncology, Houston Methodist Neal Cancer Center, Houston, TX 77030, USA; 2 Cockrell Center of Advanced Therapeutics Phase I Program, Houston Methodist Research Institute, Houston, TX 77030, USA; 3 Department of Internal Medicine, Weill Cornell Medical College, New York, NY 10021, USA; asaharia@houstonmethodist.org (A.S.); rmghobrial@houstonmethodist.org (R.M.G.); 4 Cancer Clinical Trials, Houston Methodist Research Institute, Houston, TX 77030, USA; 5 Department of Pharmacy, Houston Methodist Hospital, Houston, TX 77030, USA; goumoru@houstonmethodist.org; 6 Houston Methodist Radiology, Houston Methodist Hospital, Houston, TX 77030, USA; ktwesthart@houstonmethodist.org; 7 Section of Nephrology, Division of Internal Medicine, The University of Texas MD Anderson Cancer Center, Houston, TX 77030, USA; aabudayyeh@mdanderson.org; 8 Sherrie and Alan Conover Center for Liver Disease and Transplantation, JC Walter Jr Center for Transplantation and Houston Methodist Hospital, Houston, TX 77030, USA

**Keywords:** transplant oncology, liver transplantation, hepatocellular carcinoma, immunotherapy, immune checkpoint inhibitors, CTLA-4 inhibitors, PD-1 inhibitors

## Abstract

Systemic combination therapy of immune checkpoint inhibitors and vascular endothelial growth factors have provided the basis for improved outcomes in select patients with unresectable or metastatic hepatocellular carcinoma. However, for patients with resectable disease, surgery alone or an orthotopic liver transplant remains the standard of care. Within the realms of transplant oncology, neoadjuvant systemic therapy is currently being evaluated as a potential strategy to improve outcomes in patients with HCC. Here, we report excellent response with significant downstaging in a safe manner after neoadjuvant treatment with atezolizumab and bevacizumab in a patient diagnosed with poorly differentiated HCC. As a result of the significant response observed with safe outcomes, the patient was listed for orthotopic liver transplant (OLT) evaluation and transplanted successfully.

## 1. Introduction

Hepatocellular carcinoma is an aggressive primary liver tumor that typically evolves in the setting of chronic liver disease [1]. Risk factors for HCC are significant in cirrhotic patients due to high levels of alcohol consumption. Other risk factors include non-alcohol-associated steatohepatitis (NASH), or patients with chronic hepatitis B and C virus infection [2,3,4]. HCC comprises 75% of primary liver tumors with cholangiocarcinoma accounting for the remaining cases [1,5,6,7].

According to the world health organization GLOBOCAN database, HCC is rapidly becoming the leading cause of cancer deaths in the United States and is the fourth leading cause of cancer-related deaths worldwide. The tumor prognosis remains poor with a five-year survival rate of around 18% [8]. In the United States, the annual incidence of HCC is 6 per 100,000 [9]. Despite the low incidence rate of HCC in the US, the past two decades have seen a steady rise in incidence [9].

For early-stage HCC, the standard of care is surgery or orthotopic liver transplant (OLT) [10,11] and may be used with curative intent depending on the degree of liver dysfunction and tumor extent [12]. Unfortunately, most patients with HCC present with metastasis or locally advanced disease and would not be candidates for surgery with curative intent or transplant. For these patients, utilization of immunotherapy and VEGF inhibitors have been shown to improve outcomes after failure or intolerance to traditional chemotherapy treatment based on results from the IMbrave 150 trial. In that trial, overall survival at 12 months was 67.2% (95% CI, 61.3–73.1) with atezolizumab–bevacizumab versus 54.6% (95% CI, 45.2–64.0) with sorafenib. 

In the last decade, the transplant oncology field has been evolving and advances in locoregional and systemic therapies including immunotherapy have provided additional options for further exploration of neoadjuvant and adjuvant strategies to improve HCC resection rates [6,7,13,14,15,16,17,18,19,20,21,22,23,24,25]. We report an interesting case of a patient with HCC who developed pseudo-progression followed by an excellent response with significant downstaging after neoadjuvant treatment with atezolizumab plus bevacizumab. The patient was then listed for OLT evaluation with a plan for therapy cessation two months prior to OLT.

## 2. Case Presentation

A 66-year-old man who presented to the hospital with abdominal pain and bloating was found to have a liver mass which was determined to be hepatocellular carcinoma. The patient’s medical history was significant for benign prostatic hyperplasia (BPH), hepatitis C which was treated with interferon, and Child–Pugh class A cirrhosis. A review of systems was performed and was negative except for the presenting complaints of abdominal pain. The physical examination yielded normoactive bowel sounds with a soft non-tender abdomen with some distention without hepatosplenomegaly. The lung examination was unremarkable, vital signs were normal for this visit, and the Eastern Cooperative Oncology Group (ECOG) performance status was 1.

Liver echography and a computed tomography (CT) scan revealed a tumor mass of 5 cm in the right hepatic lobe. Portal hypertension with moderate splenomegaly and small-to-moderate ascites was also noted. The common bile duct stent (when this stent was placed, not clear) was also seen on imaging and nonspecific gallbladder wall thickening.

**Figure 1:**
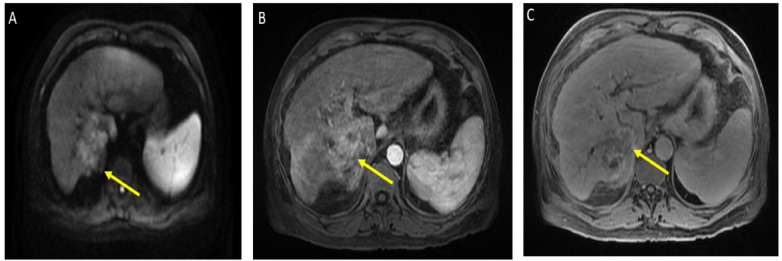
Magnetic resonance imaging of the abdomen. Limited by motion. Cirrhosis and splenomegaly were noted. Infiltrative segment 7 mass measures 6.5 × 4 cm. (**A**) Diffusion-weighted imaging (DWI) shows diffusion restriction of mass; (**B**) T1 with contrast shows vague arterial phase enhancement; (**C**) T1 post-contrast delayed phase imaging shows washout (captured at the diagnosis visit, and before the atezolizumab plus bevacizumab started).

He was admitted for tumor evaluation which revealed a poorly differentiated, multifocal, grade 3 HCC. The Liver Imaging Reporting and Data System (LI-RADS) score was 5 (for arterial enhancement, washout, and size greater than 20 mm). Mild intrahepatic biliary dilatation secondary to mass effect was also noted. The tumor was confined to the liver with clear margins, no vascular invasion, normal regional lymph nodes by imaging criteria, and no evidence of distant metastasis, as can be seen in Figure 1.

The results of liver function tests at the time of presentation included alanine aminotransferase 11 (normally 5–35) U/L, aspartate aminotransferase (AST) 17 (normally 5–30) U/L, and alkaline phosphatase (ALT) 173 (normally 25–100) U/L. Towards the end of inpatient management, the ALT level was 23, AST was 28, and alkaline phosphatase was 253. The tumor marker alpha-fetoprotein was 43.9 around the time of presentation, whereas the CA19-9 was 62 at presentation and increased to 796 by the end of management.

**Figure 2:**
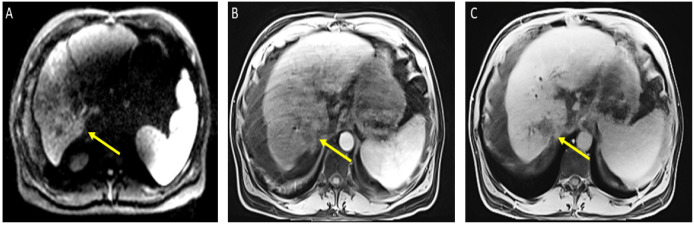
Magnetic resonance imaging of the abdomen. Cirrhosis, splenomegaly, and ascites were noted. Significant interval treatment response. Segment 7 mass has decreased in size (now 3.3 × 3 cm). (**A**) Diffusion-weighted imaging (DWI): decreased diffusion restriction of seg 7 mass; (**B**) (arterial)/(**C**) (delayed) T1 post-contrast imaging: mass is now hypovascular (captured at the follow-up visit, and 8 weeks after the atezolizumab plus bevacizumab started).

**Figure 3:**
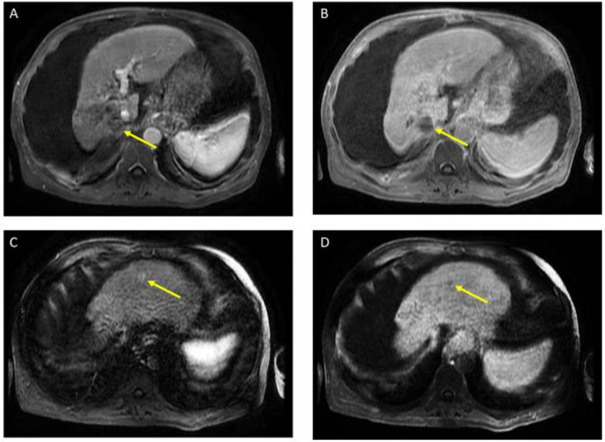
Magnetic resonance imaging of the abdomen. Post-treatment atrophy of the right hepatic lobe, cirrhosis, splenomegaly, and ascites were noted. T1 post-contrast early (**A**) and delayed (**B**) demonstrate phase unchanged Seg 7 non-enhancing 4.5 cm cavity (LR TR nonviable); T1 with contrast early (**C**) and delayed (**D**) show new 8 mm lesion in segment 2 with early enhancement and late washout (LR 4) (captured at the follow-up visit, and 6 months after the atezolizumab plus bevacizumab started).

The patient received a total of 6 cycles of atezolizumab 1200 mg intravenously plus a total of 5 cycles of bevacizumab 15 mg/kg (missed one cycle of bevacizumab) given on day 1 of a 21-day cycle. Therapy was then held for two months prior to evaluation for OLT. Follow-up MRI 8 weeks after treatment showed a significant interval treatment response with shrinkage of the segment 7 tumor, as seen in Figure 2. Additional imaging follow-up at 6 months showed post-treatment atrophy of the right lobe of the liver, stable non-enhancing segment 7 mass (LR-TR non-viable); with a new 8 mm lesion in segment 2 of the liver (LI-RADS 4 for arterial enhancement, washout, and size less than 10 mm), as can be seen in Figure 3. PET/CT scan showed no metabolic uptake in the right hepatic lobe with suspected inflammatory uptake in the common bile duct. No bone metastases were appreciated in the bone scan.

Liver biopsy is done by total hepatectomy/explant with cholecystectomy that showed classic hepatocellular carcinoma grade 3 poorly differentiated. Tumor size was 3.3 cm located on the 7th segment in the right lobe and segment 2 in the left lobe. The tumor was confined to the liver with negative margins. The Liver Imaging Reporting and Data System (LI-RADS) score was 5 with mild intrahepatic biliary dilatation s/s mass at the level of the diaphragm. The precaval long-axis diameter (LAD) with the largest node of 2.7 cm is seen at the porta hepatis. Molecular testing, which was performed for *KRAS, NRAS, BRAF*, and *HER2* were negative and the microsatellite instability (MSI) was stable.

## 3. Discussion

Here we report our utilization of neoadjuvant combination systemic therapy with atezolizumab (immunotherapy) and bevacizumab (targeted therapy) for successful tumor downstaging. Atezolizumab is a monoclonal antibody that specifically binds to PD-L1 to inhibit the interaction between PD-1 and B7.1 (i.e., CD80 receptors). PD-L1 is an immune checkpoint protein found on cancer cells that downregulates antitumor T-cell properties by binding to PD-1 and B7.1. With PD-1 and B7.1 interaction inhibited, T-cells are able to function appropriately as antitumor cells [26].

Bevacizumab is a targeted therapy that stunts tumor growth by inhibiting new blood vessels from forming [27]. Bevacizumab selectively binds circulating VEGF (vascular endothelial growth factor) and inhibits the binding of VEGF to its cell surface receptors. This inhibition directly reduces microvascular growth of tumor blood vessels and thus starves tumor tissue of blood supply. An additive effect of VEGF inhibition is the resulting increase in vascular permeability and improved delivery of chemotherapeutic agents to tumor cells further speeding up apoptosis. 

The patient in this report was treated with atezolizumab and bevacizumab combination therapy for undifferentiated hepatocellular carcinoma (grade III) prior to orthotopic liver transplant (OLT). The dose of atezolizumab and bevacizumab which was utilized for neoadjuvant treatment was extrapolated from the pivotal trial that approved this combination in the metastatic setting. After neoadjuvant treatment, imaging portrayed a significant response including the decreased size of liver mass and no observance of previously noted periductal tumor extension. He also had decreased liver enzymes corresponding with treatment response. The atezolizumab and bevacizumab therapy was held in anticipation of OLT, which was performed 8 weeks after the last dose of immunotherapy. After completion of OLT, the patient was initiated on tacrolimus 1 mg capsules twice daily and mycophenolate 500 mg tablets twice daily for immunosuppression.

**Figure 4:**
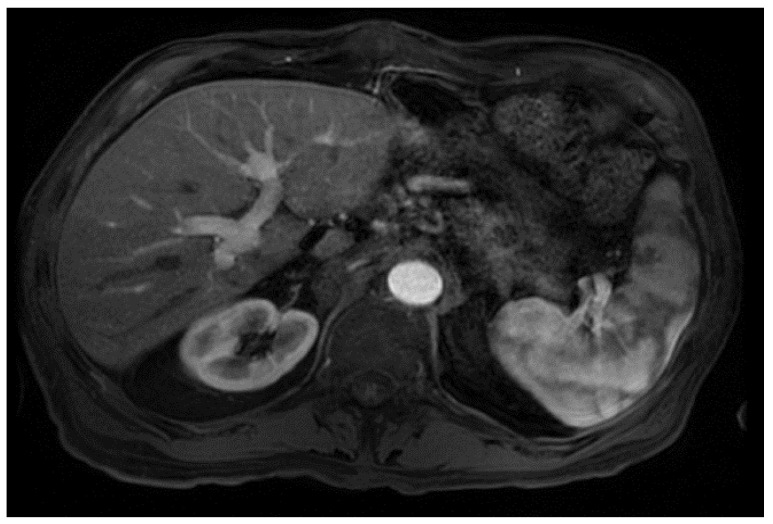
Magnetic resonance imaging of the abdomen. T1 with contrast, orthotopic liver transplant has been successfully achieved, no mass, and the impression is normal (captured at the post-transplant follow-up visit).

From a radiological perspective, the imaging plays a role in HCC diagnosis by detecting lesions, stratifying them based on standardized imaging features (LI-RADS), and providing staging information. Imaging also guides treatment by evaluating response to therapy or identifying the spread of disease; our patient had no postoperative complications or graft rejection. At his around 1-year post-transplant follow-up the patient continues to be in good health with a stable graft and no signs of cancer recurrence (Figure 4). 

In the last few years, similar therapies have been used prior to liver transplants with promising outcomes. In the case reported in the literature, pretransplant immunotherapy with pembrolizumab (anti-PDL1) was utilized for three cycles followed by curative OLT 138 days after the last dose. The patient was found to have a stable graft and no disease at 4 years post-transplant. Here we combined immunotherapy with targeted therapy as this strategy is also being evaluated by ongoing clinical trials.

Despite successful treatment in our patients and others, clinicians should be wary of the possibility of graft rejection as a result of immunotherapy. In some patients, immunotherapy induces severe graft rejection in patients due to the activation of the innate immune response. Therefore, it is important to take into consideration the timing of immune checkpoint inhibitor utilization as a crucial point in favorable outcomes. For example, in one report an adult treated with nivolumab had an OLT only eight days from the last dose of immunotherapy which likely contributed to fatal acute hepatic necrosis immediately post-surgery. Here, we report successful transplant outcomes in our patient after a prolonged period without immunotherapy (8 weeks) prior to OLT. 

Our case highlights the safety of atezolizumab and bevacizumab therapy prior to transplant while stressing the importance of timing before an organ transplant. Currently, a clinical trial is being conducted at our institution (Houston Methodist Neal Cancer Center and JC Walter Jr Center for Transplantation) to illustrate the feasibility of employing an immune checkpoint inhibitor before liver transplantation (NCT05185505) [28]. Given the paucity of data with conclusive results in this area of focus, we believe that our case report provides a foundation for prospective trials to further investigate the clinical benefit of targeting both angiogenesis and PD-L1 signaling in patients with resectable liver cancer.

## Data Availability

The data of this study that support our results are available on request from the corresponding author, Maen Abdelrahim.
